# Interoceptive accuracy moderates the response to a glucose load: a test of the predictive coding framework

**DOI:** 10.1098/rspb.2019.0244

**Published:** 2019-03-13

**Authors:** Hayley A. Young, Chantelle M. Gaylor, Danielle de Kerckhove, Heather Watkins, David Benton

**Affiliations:** Department of Psychology, Swansea University, Swansea SA2 8PP, UK

**Keywords:** interoceptive inference, predictive coding, homeostasis, glucose, mood, hunger

## Abstract

Recently, interoception and homeostasis have been described in terms of predictive coding and active inference. Afferent signals update prior predictions about the state of the body, and stimulate the autonomic mediation of homeostasis. Performance on tests of interoceptive accuracy (IAc) may indicate an individual's ability to assign precision to interoceptive signals, thus determining the relative influence of ascending signals and the descending prior predictions. Accordingly, individuals with high IAc should be better able to regulate during the postprandial period. One hundred females were allocated to consume glucose, an artificially sweetened drink, water or no drink. Before, and 30 min after a drink, IAc, heart rate (HR) and blood glucose (BG) were measured, and participants rated their hunger, thirst and mood. A higher IAc was related to lower BG levels, a decline in anxiety and a higher HR, after consuming glucose. A higher IAc also resulted in a larger decline in hunger if they consumed either glucose or sucralose. These data support the role of active inference in interoception and homeostasis, and suggest that the ability to attend to interoceptive signals may be critical to the maintenance of physical and emotional health.

## 1. Introduction

Interoception, the perception and interpretation of visceral afferent signals, underpins homeostatic functioning [1], and is an essential component in many theories of emotion [1–6]. However, interoception is no longer viewed as a ‘stimulus–response’ system, and several recent models have linked interoception and homeostatic/allostatic control to predictive coding and active inference [2,7–9].

According to these models, afferent visceral signals (i.e. interoceptive prediction errors (IPE)) update posterior predictions based upon prior beliefs (i.e. previously learned/innate expectations) about the state of the body. Descending predictions are then considered to nuance homeostatic set points that mediate physiological homoeostasis through autonomic reflexes [10]. In other words, top-down predictions are compared with experienced interoceptive states—a ‘mismatch’ results in IPE (i.e. that part of incoming interoceptive sensation not accounted for by prior expectations)—the goal is to minimize IPE [9].

Within the interoceptive system, prediction error (PE) minimization is realized, either by revising top-down predictions or by modifying the sensory signals so that they comply with the predictions (active inference) [10]. The nature of PE minimization is determined by the relative precision of ascending prediction error signals, and descending prior beliefs. Precision is the inverse variance associated with each probability distribution, thus is an index of reliability [11]. When sensory precision is high, inference is driven by sensory evidence, whereas when prior beliefs have greater precision, their influence dominates [10]. Both within and between sensory modalities, precision is continually modified. One way to optimize sensory precision is through attention [12]. For example, the ability of some individuals to perform well on tests of interoceptive accuracy (e.g. heartbeat tracking) may be due to their capacity to amplify the precision of interoceptive signals by attending to them [13].

Crucially, PE minimization is repeated throughout levels of the cortical hierarchy—messages are passed recurrently between levels [10]. At the lowest level of the hierarchy, homeostasis is maintained through the suppression of IPE by autonomic reflexes [10]. However, predictions at deeper levels embody increasingly expansive, multimodal representations of the present (and counterfactual future) interoceptive state [14]. This provides the basis for more complex forms of homeostatic regulation through the suppression of sensorimotor PE [10].

Although these frameworks have received theoretical support, to date they lack empirical verification. Nonetheless, they permit a number of testable hypotheses. Specifically, individuals who are more sensitive to interoceptive signals may (i) more readily engage innate autonomic reflexes in response to surprising interoceptive states (e.g. the difference between the expected level of glucose and the currently sensed level), and (ii) be more proficient at learning the parameters of the models that predict interoceptive state transitions. In other words, those with high interoceptive abilities may have stronger causal mappings between ascending prediction errors experienced in one sensory modality (e.g. tasting a sweet drink), and associated descending interoceptive predictions (e.g. an anticipated increase in blood glucose (BG)/gastric distension). Both mechanisms should afford those with more precise signals superior homeostatic control. Thus the primary objective of the present study was to explore the link between interoceptive accuracy (IAc) (an index of the ability to assign precision to interoceptive signals [13]), and the postprandial response to a glucose load.

Importantly, variability in the response to glucose consumption is well documented [15]. Moderating factors that have been identified include glucose intolerance [16,17], body mass index (BMI) [18], and subjective [19] and objective sensitivity to hypoglycaemia [17,20]. Although speculative, it is plausible that differences in IPE precision may mediate the influence of these factors.

In support of this suggestion individuals with lower vagal tone, measured using heart rate variability (HRV), had a greater glycaemic and appetitive response to glucose [21]. Similar effects have been reported after consuming water; an increase in HRV was related to a better mood [22]. Notably, heart rate (HR) and HRV are inversely related to interoceptive accuracy [23]. Those with higher HRV may be better able to maintain homeostasis during the postprandial period due to increased interoceptive processing (i.e. by affording more precision to ascending IPEs).

Importantly, IPEs awarded high precision are said to have privileged access to higher (possibly conscious) levels of the cortical hierarchy [24]. This potentially explains variability in the ability to consciously identify changes in glycaemia [25], a difference that has been related to the degree to which individuals experience associated subjective symptoms (e.g. hunger/mood) [26]. These data imply that although reducible IPEs at the lowest hierarchical levels (e.g. those involved in the control of BG) usually operate unconsciously, their precision may vary such that some individuals consciously experience a change in affect. Interestingly, it is proposed that irreducible IPEs underlie certain emotional states, especially anxiety [5]. Therefore, differences in IPE precision may relate to the subjective change in affect/hunger following a glucose load. A secondary aim of the present study was to test this hypothesis.

Together there is strong theoretical support for the hypothesis that differences in interoceptive accuracy should relate to the glycaemic, autonomic and subjective response to glucose. It was predicted that those with high IAc would have better homeostatic regulation. With this in mind, the present study examined the response to glucose, an artificially sweetened drink, water or no drink. The design allowed us to infer the relative contribution of PE at different levels: humeral (a change in BG), gustatory (a sweet taste) and gastric (volume of liquid consumed) signals.

## 2. Methods

### (a) Participants

The sample size was based on the expected power for a hypothesized within–between interaction. Total sample size was calculated using G*Power based on the following parameters: eight between-subject groups (four drink conditions, two IAc groups) and two within-subject levels (baseline/after taking a drink) with an expected correlation of 0.6, giving an estimated *n* of 80. With *α* = 0.05, and a two-tailed test, there was 96% power to detect a medium-sized effect (Cohen's *f*^2^ = 0.250). To be confident, 100 females between 18 and 33 years were recruited (table 1). Exclusion criteria included any metabolic or cardiovascular disorder, gastrointestinal problems, pregnancy and a diagnosis of a mood or eating disorder. BMI ranged from 17.6 to 39.7 kg m^−2^; 6.0% were underweight (BMI < 18.5), 64.0% of the sample had a normal BMI between 18.5 and 24.9, 22.0% of the sample were overweight with a BMI between 25 and 30, and the remaining 8.0% of the sample were obese with a BMI > 30. Participants refrained from drinking alcohol and physical activity within 24 h of the study, and from consuming any food and drink for at least 8 h before attending the laboratory. Testing commenced between 09.00 and 13.00.

**Table 1.** Demographic and baseline scores for those who consumed glucose, sucralose, water or nothing and those with either high of low IAc. Participants were well matched across groups; however, those with high IAc were hungrier while fasting.

	glucose		sucralose		water		nothing				
	low IAc	high IAc	low IAc	high IAc	low IAc	high IAc	low IAc	high IAc	drink	IAc	IAc × drink
age	20.3(0.9)	20.3(1.1)	20.7(2.7)	19.8(1.0)	20.1(1.1)	21.4(2.2)	20.4(1.4)	22.8(4.7)	*p* = 0.14	*p* = 0.10	*p* = 0.06
BMI	25.4(5.6)	24.3(4.4)	23.3(3.7)	21.9(2.9)	24.8(5.6)	23.7(3.8)	24.2(3.7)	25.0(4.8)	*p* = 0.21	*p* = 0.46	*p* = 0.82
fasting glucose	4.9(0.7)	5.1(0.5)	5.1(0.7)	4.9(0.8)	5.2(0.5)	5.2(0.4)	5.1(0.5)	4.9(0.5)	*p* = 0.81	*p* = 0.60	*p* = 0.58
fasting HR	75.4(9.4)	69.6(7.0)	74.9(11.8)	72.0(9.4)	74.1(13.3)	70.7(7.6)	71.4(6.7)	73.2(13.4)	*p* = 0.97	*p* = 0.20	*p* = 0.63
baseline anxiety	27.0(17.8)	40.5(23.8)	33.2(19.7)	31.5(21.0)	29.1(18.9)	29.2(22.8)	32.6(23.6)	28.8(18.4)	*p* = 0.88	*p* = 0.64	*p* = 0.41
baseline depression	36.7(19.8)	40.6(26.3)	45.2(15.0)	34.5(18.4)	36.9(14.9)	50.4(11.7)	37.2(23.7)	39.6(15.8)	*p* = 0.80	*p* = 0.56	*p* = 0.17
baseline tiredness	63.2(29.7)	61.5(27.2)	61.2(18.4)	58.3(31.5)	45.2(24.3)	67.0(17.2)	58.7(31.8)	51.5(25.3)	*p* = 0.75	*p* = 0.64	*p* = 0.29
baseline hunger	51.6(27.5)	65.1(24.4)	50.8(19.2)	69.3(26.4)	54.3(19.1)	69.7(13.6)	48.8(34.2)	52.0(26.9)	*p* = 0.45	*p* < 0.01	*p* = 0.75
baseline thirst	61.2(30.6)	74.0(27.9)	57.2(30.9)	63.9(31.0)	69.7(26.5)	86.3(10.7)	73.2(22.6)	69.7(20.5)	*p* = 0.16	*p* = 0.14	*p* = 0.65

### (b) Procedure

After providing their written informed consent, participants rated their mood, hunger and thirst. Participants had their height, weight and fasting BG measured, and conventional Ag/AgCl electrodes and transducers were applied and connected to a BIOPAC MP150 and ECG100C amplifier module (BIOPAC, USA). Participants then completed the interoception task as outlined below. Interbeat interval data were monitored throughout the interoception task with a sampling rate of 2000 Hz. The participants were then randomly allocated to receive water, sucralose, glucose or nothing. The random sequence was computer generated by H.A.Y. who produced the solutions in sequentially numbered tumblers. Participants were allocated by C.M.G. in the order they were recruited. The subjects were blind as to the nature of the drinks consumed. With the exception of hunger (*p* < 0.014), at baseline the groups were well matched for subjective ratings, interoception, fasting BG and BMI (table 1). Participants were given 5 min to consume the beverage, following which they relaxed (reading of watching TV) for 30 min, before they again rated their mood, hunger and thirst, completed the heartbeat perception task, and a BG measurement was taken. HR was recorded for a second time. Finally, after 60 min another BG measurement was taken.

### (c) Test drinks

Each drink was 500 ml provided in a clear plastic tumbler. The glucose drink contained 75 g of glucose dissolved in water. The sugar-free beverage was sweetened with sucralose to produce a similar sweetness to the other drink, which was confirmed during previous experiments [21]. These two drinks contained 10 ml of lemon juice to increase palatability. An equal volume of plain water was consumed in the water condition. Participants who consumed nothing were unaware that other participants had consumed a drink.

### (d) Interoceptive accuracy

The heartbeat perception task was performed using the mental tracking method [27] with intervals of 30, 35, 40, 45 and 50 s that were separated by 30 s resting periods. During each trial R-R intervals were recorded and participants were asked to silently count their heartbeats without the use of an exteroceptive aid (such as taking one's pulse). At the end of each period participants reported the number of counted heartbeats. The participants were not informed about the length of the counting phases nor about the quality of their performance. The transformation 1 − ∑ (abs(actual − reported))/(actual) was used to calculate heartbeat tracking scores. These scores were then averaged to form a mean heartbeat tracking score (IAc). The interoception score varied between 0 and 1 with a higher score indicating better accuracy. The internal consistency of this measure was excellent: Cronbach's *α* = 0.96. This heartbeat tracking task is a standard measure used to assess the accuracy of the ability to detect interoceptive signals and was chosen as it may specifically measure an individual's ability to selectively attend to interoceptive signals [13].

### (e) Blood glucose

BG was monitored from finger pricks using an ExacTech sensor (Medisense Britain Limited) with an enzymic method, coupled with microelectronic measurement, which has been shown to be accurate [28].

### (f) Mood, hunger and thirst

Participants were asked to describe the way they felt ‘at that moment’ using visual analogue scales (VAS) with pairs of adjectives at the ends of 100 mm lines: composed/anxious, elated/depressed, tired/energetic, not at all hungry/extremely hungry and not at all thirsty/extremely thirsty [29].

### (g) Body mass index

Body mass was measured using an electronic scale (Kern KMS-TM, Kenr and Sohn GmbH, Germany) that took 50 assessments over a 5 s period and produced an average value. Height was measured using a portable stadiometer.

### (h) Control of the proportion of type 1 errors

The present study examined effects on seven dependant variables therefore the potential of detecting false positives was controlled using Benjamini and Hochberg's false discovery rate (FDR). The FDR was controlled at *δ* = 0.05. (electronic supplementary material, table S1). Where significant interactions did not reach this threshold this is indicated in the text. Confidence intervals for simple effects were adjusted using the Bonferroni correction.

## 3. Results

### (a) Descriptive results

A one-sample *t*-test confirmed that the overall score for IAc was above chance level (mean = 0.64, s.d. = 0.19), *t* = 33.06, *p* < 0.001). Those who scored above this level were considered to have high IAc, whereas those below were considered to have low IAc.

### (b) The effect of interoceptive accuracy on changes in blood glucose

Initially it was considered whether the effect on BG of consuming glucose, rather than sucralose, water or nothing, varied according to individual differences in IAc. A 4 (drink: glucose, sucralose, water, nothing) × 2 (IAc: high, low) × 3 (time: fasting BG, 30 min BG, 60 min BG) repeated-measures ANOVA was conducted. BMI and age were considered covariants. Neither age (*F* = (1, 90) = 0.034, *p* = 0.855, $\eta_{p}^{2}=0.001$) nor BMI (*F* = (1, 90) = 3.523, *p* = 0.064, $\eta_{p}^{2}=0.038$) contributed significantly to the model.

**Figure 1.**
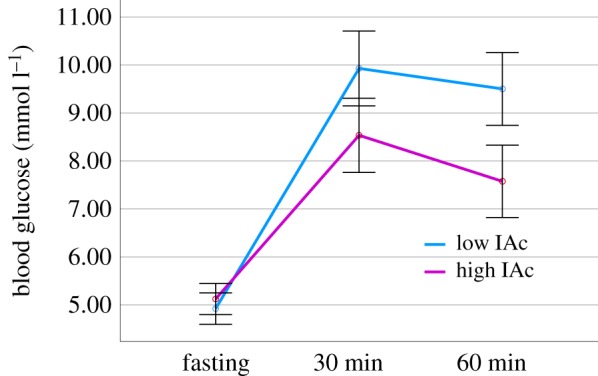
The glycaemic response to consuming glucose in those with high or low IAc. *n* = 30. After both 30 (*p* < 0.013) and 60 (*p* < 0.001) minutes, those with low IAc had significantly higher blood glucose levels than those with high IAc. (Online version in colour.)

As expected the interaction time × drink was significant (*F* = (6,180) = 38.705, *p* < 0.001, $\eta_{p}^{2}=0.563$): participants who consumed glucose had significantly higher BG levels after both 30 and 60 min compared to all other drinks (all *p* < 0.001). The interaction time × drink × IAc also reached significance (*F* = (6,180) = 2.832, *p* < 0.012, $\eta_{p}^{2}=0.086$). In those who consumed sucralose, water or nothing, BG did not differ at any time point depending on IAc (all *p* > 0.285). Similarly, IAc did not influence fasting BG across the entire sample (*p* = 0.841) or in the group who drank glucose (*p* = 0.386). However, after 30 min those with low IAc had higher BG levels if they consumed glucose (figure 1) (*p* < 0.013). The effect was similar after 60 min (*p* < 0.001). These findings suggest that individuals with higher IAc may be better able to regulate their glycaemic response to glucose.

### (c) The effect of interoceptive accuracy and glucose on heart rate

**Figure 2.**
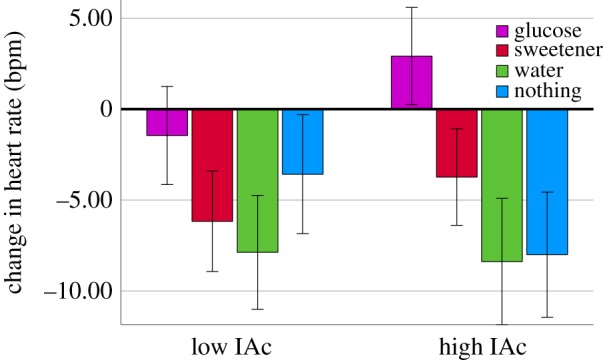
Change in HR from before to after consuming either glucose, sucralose, water or nothing in those with high or low IAc. *n* = 100. In those with high IAc, consuming glucose resulted in a higher HR (*p* < 0.033). (Online version in colour.)

Next it was considered whether consuming glucose, rather than sucralose, water or nothing, influenced HR and whether this varied according to individual differences in IAc. A 4 (drink: glucose, sucralose, water, nothing) × 2 (IAc: high, low) × 2 (time: fasting HR, 30 min HR) repeated-measures ANOVA was conducted. BMI and age were considered covariants. Neither BMI (*F* = (1, 90) = 0.070, *p* = 0.792, $\eta_{p}^{2}=0.001$) nor age (*F* = (1, 90) = 0.443, *p* = 0.508, $\eta_{p}^{2}=0.005$) were related to HR. There was a significant time × drink interaction (*F* = (3, 90) = 13.206, *p* < 0.001, $\eta_{p}^{2}=0.306$), reflecting a decline in HR, from T1 to T2, in those who consumed sucralose (*p* < 0.001), water (*p* < 0.001) or nothing (*p* < 0.001); the effect was absent in those who drank glucose (*p* = 0.448). There was also a significant time × drink × IAc interaction (*F*_3,90_ = 2.989, *p* < 0.035, $\eta_{p}^{2}=0.091$). IAc did not influence the autonomic response to consuming sucralose, water or nothing (figure 2). However, in those who drank glucose, a higher IAc was associated with an increase in HR after the drink (*p* < 0.033). Conversely, this effect was absent in those with low IAc (*p* = 0.288).

### (d) The effect of interoceptive accuracy and glucose on mood

**Figure 3.**
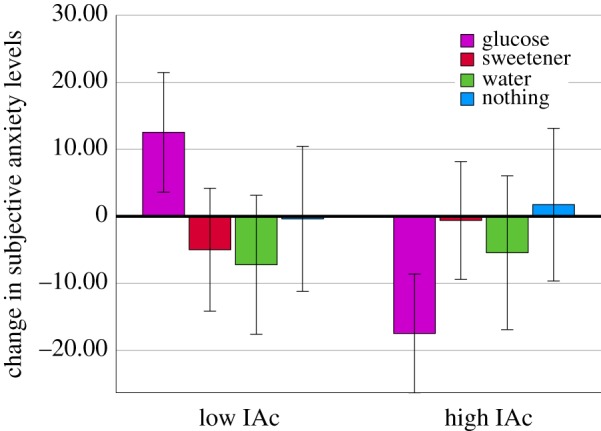
Change in anxiety from before to after consuming either glucose, sucralose, water or nothing in those with high or low IAc. *n* = 100. In those who consumed glucose, those with high IAc experienced a decline in ANX (*p* < 0.001). This effect was reversed in those with low IAc who experienced an increase in anxiety after consuming glucose (*p* < 0.006). (Online version in colour.)

From the interoceptive inference perspective, emotions are motivating signals that arise as a result of a discrepancy between expected and actual body states (interoceptive prediction error). If correct then IAc should relate to the affective response to glucose (a homeostatic challenge that produces PE). Initially effects on ratings of anxiety (ANX) were considered. A 4 (drink: glucose, sucralose, water, nothing) × 2 (IAc: high, low) × 2 (time: fasting anxiety, 30 min anxiety) repeated-measures ANOVA was conducted. BMI and age were considered covariants. Neither BMI (*F* = (1, 90) = 1.552, *p* = 0.216, $\eta_{p}^{2}=\;0.017$) nor age (*F* = (1, 90) = 2.203, *p* = 0.141, $\eta_{p}^{2}=0.024$) were related to ANX. However, the Time × Drink × IAc interaction was significant (*F* = (3, 90) = 6.431, *p* < 0.001, $\eta_{p}^{2}=0.177$). In those who consumed glucose, those with high IAc experienced a decline in ANX (*p* < 0.001). Interestingly, this effect was reversed in those with low IAc who experienced an increase in anxiety after consuming glucose (*p* < 0.006). IAc did not influence anxiety in any of the other groups (all *p* > 0.169) (figure 3). Assuming those high in IAc are better able to minimize PE, this may explain their reduction in anxiety.

The analysis was repeated for ratings of depression (DEP). Older participants were more depressed (*F* = (1, 90) = 5.670, *p* < 0.019, $\eta_{p}^{2}=0.059$). The effect of BMI was also significant (*F* = (1, 90) = 4.193, *p* < 0.044, $\eta_{p}^{2}=0.044$). However, none of the interactions involving drink or IAc reached significance: time × drink (*F* = (3, 90) = 0.036, *p* = 0.991, $\eta_{p}^{2}=0.001$) and time × drink × IAc (*F* = (3, 90) = 1.063, *p* = 0.369, $\eta_{p}^{2}=0.034$).

Similarly, when tiredness was considered, only age reached significance (*F* = (1, 90) = 8.007, *p* < 0.006, $\eta_{p}^{2}=0.082$); older participants were more tired. BMI did not relate to energy levels (*F* = (1, 90) = 0.847, *p* < 0.360, $\eta_{p}^{2}=0.009$), and neither were any of the interactions involving drink or IAc: time × drink (*F* = (3, 90) = 1.404, *p* = 0.247, $\eta_{p}^{2}=0.045$) and time × drink × IAc (*F* = (3, 90) = 0.484, *p* = 0.694, $\eta_{p}^{2}=0.016$).

### (e) The effect of interoceptive accuracy and glucose on hunger and thirst

In the context of interoception, hunger and thirst are arguably the most motivationally relevant subjective feelings. A 4 (drink: glucose, sucralose, water, nothing) × 2 (IAc: high, low) × 2 (time: fasting anxiety, 30 min anxiety) repeated-measures ANOVA was conducted. BMI and age were considered covariants. Age contributed significantly (*F* = (1, 90) = 4.093, *p* < 0.046, $\eta_{p}^{2}=0.044$), but BMI did not (*F* = (1, 90) = 3.589, *p* < 0.061, $\eta_{p}^{2}=0.038$).

**Figure 4.**
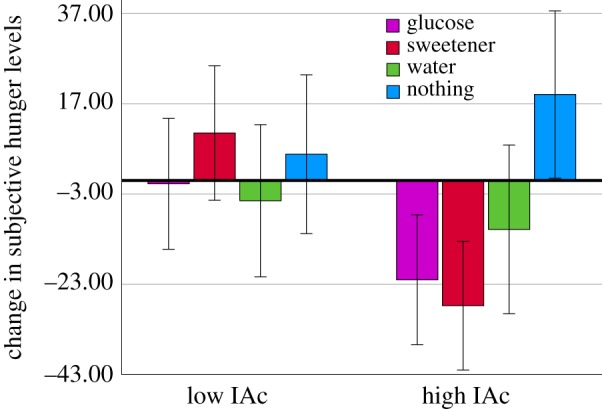
Change in hunger from before to after consuming either glucose, sucralose, water or nothing in those with high or low IAc. *n* = 100. Those with high, but not low, IAc had a decline in hunger after glucose (*p* < 0.003) and sucralose (*p* < 0.001) (*p* = 0.921 and *p* = 0.163 respectively in those with low IAc). In addition, those with high, but not low, IAc had an increase in hunger after nothing (*p* < 0.044) (*p* = 0.512 in those with low IAc). No effects were observed in those who consumed water (high IAc *p* = 0.252, low IAc *p* = 0.598). (Online version in colour.)

The drink × time interaction was significant (*F* = (3, 90) = 3.156, *p* < 0.029, $\eta_{p}^{2}=0.095$); those who drank glucose (*p* < 0.031) experienced a significant decline in hunger. In addition, the time × drink × IAc interaction was also significant (*F* = (3, 90) = 3.381, *p* < 0.022, $\eta_{p}^{2}=0.101$); however, as the FDR threshold for this interaction was *p* < 0.021 this effect should be interpreted with caution (electronic supplementary material, table S1). Nonetheless, those with high, but not low, IAc had a decline in hunger after glucose (*p* < 0.003) and sucralose (*p* < 0.001) (*p* = 0.921 and *p* = 0.163 respectively in those with low IAc). In addition, those with high, but not low, IAc had an increase in hunger after nothing (*p* < 0.044) (*p* = 0.512 in those with low IAc). No effects were observed in those who consumed water (high IAc *p* = 0.252, low IAc *p* = 0.598) (figure 4).

Similar effects were seen when thirst was considered. Neither the effect of BMI nor the effect of age was significant. However, the time × drink interaction was significant (*F*_3,90_ = 4.387, *p* < 0.006, $\eta_{p}^{2}=0.128$). After a drink, those who consumed water were less thirsty than those who consumed nothing (*p* < 0.007). The time × drink × IAc interaction was significant (*F* = (3, 90) = 3.333, *p* < 0.023, $\eta_{p}^{2}=0.100$). Participants who consumed nothing were thirstier than those who consumed glucose (*p* < 0.035), sucralose (*p* < 0.026) and water (*p* < 0.002), but only if they had high IAc (*p* = 0.613, *p* = 0.821, *p* = 0.158 respectively in those with low IAc).

### (f) The effect of changes in BG on changes in interoceptive accuracy

Hitherto, we have examined differential responses in people with high and low baseline interoceptive accuracy. However, an important aspect of interoceptive inference is the circular causality between states of the body and inferred bodily states. One aspect of this is that BG, in and of itself, may change IAc. Partial correlation analysis (controlling for age and BMI) revealed an interesting pattern of results (table 2). Sixty minutes after glucose consumption those with higher IAc had lower BG levels (*r* = −0.379, *p* < 0.047). A positive correlation was observed between an increase in BG after 30 min and a change in IAc but this effect did not reach significance (*r* = 0.344, *p* < 0.073). Conversely, in both the water (*r* = −0.569, *p* < 0.013 after 60 min) and nothing conditions (*r* = −0.572, *p* < 0.013 after 30 min, *r* = −0.523, *p* < 0.026 after 60 min) negative correlations between changes in BG and changes in IAc were observed. Interestingly, despite a similar decline in BG levels no correlations were observed in those who consumed sucralose.

**Table 2.** Results from the partial correlation analysis that assessed the associations between change in blood glucose and changes in heartbeat perception.

		glucose			sucralose			water			nothing		
	fasting	Δ 30-F	Δ 60-F	Δ 60–30	Δ 30-F	Δ 60-F	Δ 60–30	Δ 30-F	Δ 60-F	Δ 60–30	Δ 30-F	Δ 60-F	Δ 60–30
mean	5.08	4.19	3.66	−0.53	−0.04	−0.19	−0.14	−0.52	−0.74	−0.21	−0.34	−0.44	−0.10
range	3.70–6.90	0.20–10.20	−0.50–11.60	−8.30–6.30	−1.40–1.80	−1.60–1.00	−1.80–1.10	−1.30–0.60	−2.10–0.30	−1.00–0.40	−1.30–0.40	−1.90–0.50	−1.50–1.30
F IAc	0.046	−0.173	−0.379*	−0.205	0.210	0.222	−0.037	0.253	0.202	0.064	−0.455^+^	−0.097	0.191
Δ IAc	0.048	0.344^+^	0.123	−0.192	−0.119	−0.170	−0.029	−0.399	−0.569*	−0.536*	−0.572*	−0.523*	−0.197
F HR	−0.041	0.463*	0.438*	0.002	0.093	0.097	−0.018	−0.454^+^	−0.364	−0.118	0.392	0.354	0.120
Δ HR	−0.097	−0.120	−0.184	−0.067	0.300	0.329	−0.040	0.531*	0.613*	0.463^+^	−0.037	−0.173	−0.164

*p < 0.050.

^+^*p* < 0.08.

## 4. Discussion

The objective of the present study was to empirically test predictions from the active inference framework within the interoceptive domain. Key findings were that after consuming glucose individuals with high interoceptive accuracy (IAc) had lower BG levels, a decline in anxiety (but not depression or fatigue), and greater autonomic nervous system (ANS) reactivity. Those with high IAc also had a decline in hunger after consuming either glucose or sucralose. Taking IAc as an index of the capacity to use attention to prioritize interoceptive signals [13], these findings suggest that high sensory precision may facilitate homeostatic and affective regulation during the post-prandial period.

This is the first report that those with higher IAc have a greater autonomic responsiveness to a glucose challenge (figure 2). However, a number of studies have reported greater autonomic reactivity in individuals with high IAc more generally. For example, those high in IAc experienced stronger HR responses to a range of emotionally pleasant and unpleasant stimuli [30,31]. In addition, differences in IAc may account for variation in responses to other homeostatic challenges. Herbert *et al.* [32] considered whether IAc modified the self-regulatory response to a physical load (self-paced cycling). Interestingly, good heartbeat perceivers showed a smaller increase in HR, stroke volume and cardiac output. However, they also covered a significantly shorter distance, an effect that correlated positively with the autonomic changes [32]. This suggests that the reduced autonomic response in good heartbeat perceivers might be explained by less physical effort—a high sensory precision may have facilitated behavioural self-control of workload through the propagation of IPEs to higher levels of the hierarchy. Future research where participants are not afforded the opportunity to self-pace is required to determine whether comparable increases in autonomic activity to those in the present study are observed.

A consideration is that the present study measured autonomic reactivity within the cardiac domain, and it remains to be tested whether similar effects would be observed in other domains (e.g. vagal efferent innervation of the pancreas). Nonetheless, compensatory cardiovascular changes following a glucose load are necessary to prevent a postprandial fall in blood pressure [33]. Therefore, communication between the cardiovascular and glucoregulatory systems is a physiological requirement, and changes in autonomic functioning within the cardiac domain represent an important component of the homeostatic response.

An important finding of the present study was that individuals with lower IAc had higher BG levels (figure 1), suggesting that such individuals have poorer homeostatic regulation. The first possible explanation for this finding is that a high sensory precision makes good heartbeat perceivers more sensitive to ascending IPEs (e.g. an unexpected change in BG). This could facilitate homeostasis through the engagement of reflexes at low levels of the cortical hierarchy (e.g. vagally mediated secretion of insulin [34]). Indeed it has been argued that impaired glucose homeostasis may be caused by initial defects in glucose sensing [35].

However, a critical question concerns the connection between conscious heartbeat perception accuracy, with neural correlates in the insula and prefrontal cortex [36], and unconscious homeostatic control, occurring mainly in the brainstem and the hypothalamus [35]. Individuals at rest are not usually aware of their heartbeat—heartbeat tracking tasks ‘require’ individuals to direct their attention consciously towards this interoceptive modality. Given that homeostatic control proceeds without the need for conscious attention it is interesting that the two should be related.

From a behavioural perspective, recent frameworks have differentiated between different interoceptive dimensions. Originally, Garfinkel *et al.* [37] argued that (i) interoceptive accuracy (objective performance), (ii) interoceptive metacognitive awareness (confidence–accuracy correspondence) and (iii) interoceptive sensibility (self-evaluation) should be considered independently. This has been extended to include (iv) afferent signal (e.g. baroreceptor activity/heart evoked potential), (v) preconscious impact of interoceptive signals on conscious processing (e.g. presenting stimuli at different parts of the electrocardiogram) and (vi) executive (switching between modalities) [38].

Using such frameworks comparable paradigms can be developed to assess interoceptive abilities *across* domains. For example, IAc (heartbeat tracking) might be akin to the accuracy of estimated BG levels [25], a change in BG might be analogous to changes in the firing rate of action potentials from the baroreceptors [39], while the subjective response to a change in BG (figure 3) may align to the preconscious impact of cardiovascular signals on the subjective interpretation of stimuli [40].

However, it remains uncertain to what degree interoceptive dimensions at different levels interact. For example, conscious attention to the heartbeat increased the amplitude of the heart evoked potential [41]; a measure thought to reflect the strength of the afferent signal [38], and positively related to performance on the heartbeat tracking task [42]. Within the glucoregulatory domain, variability in BG estimation accuracy was related to the degree to which individuals subjectively perceived a change in mood [26]. Furthermore, providing conscious information about BG levels improved the ability to accurately estimate current BG levels [43]. In addition, the cephalic phase response aptly demonstrates circular causality between expected bodily states, and the current state of the body [44]. These interoceptive beliefs may operate at conscious or unconscious levels [45], alter homeostatic states [44], and are in turn learned from previous interoceptive sensations [46]. Within the cardiac domain, the influence of preconscious baroreceptor signalling on memory was modulated by participant's conscious performance on a heartbeat perception test [47]. Additionally, self-reported confidence correlated with heartbeat perception, but only in those with high IAc [37]. Together, these findings suggest that *within* domains interoceptive dimensions occurring at different degrees of consciousness are somewhat interdependent. Such data might explain the present observation that a measure of conscious interoceptive accuracy (IAc) was related to the unconscious homeostatic (figure 1) and conscious subjective (figures 3 and 4) responses to glucose.

Few studies have assessed interoception *across* domains simultaneously; generally moderate relationships across axes are reported. For example, Herbert *et al.* [48] found an inverse relationship between IAc and the amount of water a person could consume until reaching the point of individually perceived fullness. Conversely, Garfinkel *et al.* [49] recently found no association between cardiac and respiratory measures of IAc (i.e. task performance). However, interoceptive metacognitive awareness generalized across these domains.

Interestingly, there is also anatomical and neuroimaging evidence that the brain tracks or integrates different interoceptive signals in similar regions including insula, somatosensory cortices, cingulate, amygdala, thalamus and brainstem [9,50]. For example, peripheral BG levels have been linked to changes in insula activity [51,52], an area of the brain often associated with heartbeat perception [53], and thought to play a role in registering IPE [54]. Together with the present findings these data suggest that there may be a general interoceptive sensitivity across cardiovascular and glucoregulatory domains. Specifically, not only did fasting IAc relate to subsequent changes in BG, but after consuming water or not drinking a decline in BG was associated with an increase in IAc (table 2). Taken in the context of the present literature suggesting that interoceptive processes associate *across* and *within* modalities, these findings lend support to a key aspect of predictive coding—that precision represents a ‘common currency’ across perceptual domains, at every level of the hierarchy [10].

A second interpretation of the present findings is that the gustatory properties (e.g. sweet taste) of the drink could have induced beliefs about interoceptive changes (e.g. an anticipated increase in BG). This may have contributed to a cephalic phase response that facilitated homeostatic control. Prior beliefs may be innate or learned, therefore high IAc may facilitate the acquisition of generative models driving cephalic responses. That those with high IAc had a decline in hunger after both glucose and sucralose is consistent with this interpretation (figure 4). Indeed there is evidence that gustatory and cardiovascular information is integrated within the insula [55]. However, there is controversy over whether the sensation of sweetness alone is an effective stimulus for the cephalic phase response [56], with no effects observed after modified sham feeding with sucralose [57], leading to claims that there are responders and non-responders [58]. The present findings suggest that individual differences in the ability to prioritize interoceptive signals may be an important moderator of the cephalic response; an important avenue for future research.

A final observation was that those with higher IAc reported a decline in anxiety if they consumed glucose; the opposite pattern was observed in those with lower IAc (figure 3). Recent proposals argue that a negative emotional valence results from irreducible free energy (the sum total of PEs), whereas a reduction in free energy produces positive affect [59]. Emotional arousal is hypothesized to depend on interoceptive precision [13]. Indeed, individuals high in anxiety tend to perform better on heartbeat perception tasks [5,60,61]. The present observation that after consuming glucose those high in IAc reported a decline in anxiety may be explained by their ability to effectively minimize PEs. This is supported by their greater autonomic reactivity and better glycaemic regulation (figures 1 and 2). An increase in anxiety in those with lower IAc could be due to an increase in irreducible free energy. Future research should consider the dimensional nature of affect, for example by dissociating changes in valence and arousal.

Although IAc was related to changes in anxiety, no effects were observed when depression or tiredness was considered. Interestingly, fatigue and depression may result from a chronic inability to maintain homeostasis [14], whereby individuals become ‘locked in’ to an energy-inefficient internal model [62]. Accordingly, this may result from poorly calibrated precision estimates due to aberrant hyperpriors (prior expectations about precision), and consequently an insensitivity to IPEs. On the other hand, due to a loss of prior precision, those with anxiety may remain responsive to peripheral feedback [63]. This might explain why ratings of depression and tiredness were not altered by the nature of the drink. As mood is thought to represent a hyperprior over precision, future research might investigate how underlying interoceptive computations present biologically in response to other homeostatic challenges.

The limitations of the present study should be considered. First, the validity of the heartbeat counting task has been questioned due to the possibility that participants may base their counts on beliefs about HR [64]. Therefore, future research should seek to replicate these findings using a more robust heartbeat discrimination task based on the method of constant stimuli [23]. In addition, it has previously been considered that only those above 0.85 on this heartbeat counting test be considered to have high IAc [65]. This study should be replicated using a preselected sample of participants scoring 0.85 or higher. The design of the study meant that participants attended the laboratory having fasted for 8 h, and in some cases received no drink. This meant that participants were fasted for different lengths of time. As food deprivation has been shown to influence interoceptive awareness [66] this may not have been an entirely neutral control condition. Ratings of hunger were correlated with IAc at baseline (table 1), so it is possible that absolute ratings of hunger may have influenced the results—those who arrived hungrier may have had higher IAc, and subsequently a larger decline in hunger. However, selecting participants within the glucose condition so that they were matched on hunger at baseline did not alter the pattern of results (baseline: high IAc 62.5(4.8), low IAc 63.4(4.8); after: high IAc 54.8(6.5), low IAc 40.5(6.5)). A factor that could limit the generalizability of the results of this study is that, while 100 participants were recruited, the possibility exists that the study was underpowered to detect between-subject effects. In addition, the sample comprised only young college students and future research may consider different populations who may have more difficulties with regulation of their BG levels.

## 5. Conclusion

In conclusion, the present study provides empirical data in support of the role of interoceptive inference in the control of homeostasis. The finding that after a glucose load a better interoceptive accuracy (IAc) was related to lower BG levels, a decline in rating of anxiety and larger modifications of ANS functioning is consistent with the view that those with higher IAc are better able to assign precision to interoceptive signals [13]. It is plausible that such individuals are able to increase precision in interoceptive systems more generally, lending support to the contention that there is a general sensitivity for interoceptive processes across modalities. Recent conceptualizations of ‘health’ emphasize resilience and the capacity to adapt to daily challenges [67], including homeostatic challenges [68]. The present data indicate that the ability to attend to interoceptive signals may be critical to this process. It has been argued that differences in interoception drive symptom inter-correlation across psychiatric conditions, and thus give rise to the hypothetical ‘p factor’ [69]. Given that the maintenance of homeostasis is considered a core function of interoceptive inference, it is recommended that such proposals be extended to also encompass physical health.

## Supplementary Material

Table S1. False discover procedure

## Data accessibility

Data available at https://osf.io/w6np3/.
