# Supplementary material for: Interoceptive accuracy moderates the response to a glucose load: a test of the predictive coding framework
Source: Proc Biol Sci. 2019 Mar 13;286(1898):20190244. doi: 10.1098/rspb.2019.0244 (PMC6458315; doi:10.1098/rspb.2019.0244)
Supplement: Table S1. False discover procedure [file rspb20190244supp1.pdf]

# Interoceptive Accuracy Moderates the Response to a Glucose Load: A Test of the Predictive Coding Framework.

Hayley A Young et al. Swansea University, 2019

Proceedings of the Royal Society B: Biological Sciences. DOI

10.1098/rspb.2019.0244

Supplementary material

**Table S1. False discover procedure**

| RANK(j) | P - VALUE | (j/m) x δ | REJECT H0? |
|---|---|---|---|
| 1 | 0.001 | 0.007 | 1 |
| 2 | 0.012 | 0.014 | 1 |
| 3 | 0.022 | 0.021 | 0 |
| 4 | 0.023 | 0.028 | 1 |
| 5 | 0.035 | 0.035 | 1 |
| 6 | 0.369 | 0.042 | 0 |
| 7 | 0.694 | 0.050 | 0 |
